# Should video laryngoscopy or direct laryngoscopy be used for adults undergoing endotracheal intubation in the pre-hospital setting? A critical appraisal of a systematic review

**DOI:** 10.1002/14651858

**Published:** 2023-06-02

**Authors:** Elspeth Pennington, Steve Bell, James E Hill

**Affiliations:** Research Paramedic, North West Ambulance Service NHS Trust; Consultant Paramedic, North West Ambulance Service NHS Trust; University of Central Lancashire, Colne, Lancashire

**Keywords:** Airway management, endotracheal intubation, Laryngoscopy, Pre-hospital, Video laryngoscopy

## Abstract

The safety and utility of endotracheal intubation by paramedics in the United Kingdom is a matter of debate. Considering the controversy surrounding the safety of paramedic-performed endotracheal intubation, any interventions that enhance patient safety should be evaluated for implementation based on solid evidence of their effectiveness. A systematic review performed by Hansel and colleagues (2022) sought to assess compare video laryngoscopes against direct laryngoscopes in clinical practice. This commentary aims to critically appraise the methods used within the review by Hansel et al (2022) and expand upon the findings in the context of clinical practice.

## Introduction

Endotracheal intubation (ETI) by paramedics within the United Kingdom (UK) is a contentious topic with its place within the scope of paramedic practice subject to varying opinions regarding the safety and utility of the procedure (Gregory, 2015). Much of this debate stems from the perceived level of skill and competence required to undertake the procedure and the associated education, training, and assessment processes to determine practitioner competency (Gregory, 2015). It is also recognised that for the majority of UK paramedics exposure to undertaking ETI in pre-hospital clinical practice is limited and therefore robust mechanisms are necessary to monitor and ensure on-going clinical competency (Gowens et al, 2018).

Given the contention over the safety of ETI by paramedics (Pallin, 2018), any innovations which add to patient safety should be considered for introduction based upon robust evidence of effectiveness. Video laryngoscopy is one such potential innovation with the addition of electronic camera technologies to laryngoscopes offering the practitioner improved views of the glottis during ETI with the inference of improving success rates associated with the procedure (Chemsian et al 2014). The systematic review performed by Hansel and colleagues (2022) sought to assess to compare video laryngoscopes against direct laryngoscopes in clinical practice and provides a contemporary and thorough review of video verses direct laryngoscopy in adult patients. From the outset it should be recognised that the systematic review does not have a pre-hospital or paramedic focus and instead considers the evidence base from all fields of practice associated with ETI.

### Aim of commentary

This commentary aims to critically appraise the methods used within the review by Hansel et al (2022) and expand upon the findings in the context of clinical practice.

### Methods of the systematic review by Hansel et al (2022)

A comprehensive, peer-reviewed, multi-database literature search was carried out from January 2015 to 27 February 2021, with this start date having been chosen due to previous knowledge that no papers on the topic were available before this date. There were no restrictions on language or publication status. Only randomised controlled trials (RCTs), of parallel or cross-over design, comparing the use of any model of video laryngoscopy versus direct laryngoscopy with a Macintosh blade, with participants aged 16 years and older, in any setting, were included. Studies focusing on awake tracheal intubation, simulation not involving human participants, and those using manikins or cadavers were excluded. Studies using optical stylets, flexible fibreoptic intubating devices, tracheal tubes with an integrated camera, and McCoy or Miller direct laryngoscopy blades were all also excluded.

A thorough screening process of title and abstracts and then full-text articles was undertaken by two reviewers independently, with disagreements being resolved by discussion, or adjudication of a third reviewer. A comprehensive assessment of bias was undertaken by two reviewers using Higgins’ (2011) Risk of Bias 1 tool. Meta-analysis was undertaken using a random effects model due to pre-perceived study level heterogeneity. For all binary outcomes the relative risk (RR) and corresponding 95% confidence intervals (95% CI) were calculated. Heterogeneity was examined using the I^2^ statistic.

The key findings from the review were split into three comparison groups comparing the following different styles of video laryngoscopy devices with direct laryngoscopy:

- Macintosh-style video laryngoscopy Vs direct laryngoscopy
- Hyper-angulated video laryngoscopy Vs direct laryngoscopy
- Channelled video laryngoscopy Vs direct laryngoscopy

### Results of the systematic review by Hansel et al (2022)

The search strategy identified 2344 records after duplicates were removed. After full screening 222 RCTs were included in the review (158 new studies and 64 studies included from the previous version of the review). The included studies were from several different countries across the world and of a mixture of high, middle and low incomes. Most of the studies were from the theatre setting with only 6 from the pre-hospital setting and only half of these pre-hospital studies had data extracted from them for the meta-analysis due to having marked outliers for the key outcome of failed intubation rates.

There was moderate certainty evidence that Macintosh-style video laryngoscopy may provide a clinical and statistically significant reduction in risk of failed intubation (RR 0.41 95% CI: 0.26 to 0.65), hypoxaemia (RR 0.72 95% CI: 0.52 to 0.99) and Cormack-Lehane views Grade 3 or 4 (RR 0.38, 95% CI: 0.29 to 0.48). There was low certainty evidence that Macintosh-style video laryngoscopy increases the risk of successful first attempt compared to direct laryngoscopy (RR 1.05, 95% CI:1.02 to 1.09). There was low to very low evidence that there was no evidence of effect for both oesophageal intubation and dental trauma.

When comparing hyperangulated video laryngoscopy to direct laryngoscopy, there was moderate quality evidence that there was a clinically and statistically significant reduction in the risk of failed intubation (RR 0.51, 95% CI: 0.34 to 0.76), oesophageal intubation (RR 0.39, 95% CI: 0.18 to 0.81) and Cormack-Lehane views Grade 3 or 4 (RR 0.15, 95% CI: 0.10 to 0.24). There was low certainty evidence that utilising hyperangulated video laryngoscopy may slightly increase the chances of a successful first attempt (RR 1.03 95% CI: 1.00 to 1.05). There was low to very low evidence that there was no evidence of difference for dental trauma and hypoxaemia.

When comparing channelled video laryngoscopy compared to direct laryngoscopy there was moderate quality evidence that there was a clinical and statistically significant reduction in risk of failed intubation (RR 0.43, 95% CI: 0.30 to 0.61), hypoxia (RR 0.25, 95% CI: 0.12 to 0.50) and Cormack-Lehane views Grade 3 or 4 (RR 0.14, 95% CI: 0.09 to 0.21). There was very low certainty evidence that channelled video laryngoscopy compared to direct laryngoscopy increased the risk of successful first attempt (RR 1.10 95% CI: 1.05 to 1.15). There was low to very low evidence that there was no evidence of difference for dental trauma and oesophageal intubation. Due to substantial heterogeneity time for tracheal intubation was not meta-analysed for any comparison.

### Subgroup analyses

For the subgroup analysis the post hoc decision was made to compare all techniques combined compared to direct laryngoscopy for the outcome of failed intubation. There was a statistically significant reduction in risk of failed intubation for difficult airway features (RR 0.32, 95% CI 0.23 to 0.44; I^2^ = 9%) compared to no difficulty (RR 0.54, 95% CI 0.38 to 0.78; I^2^ = 23%). There was a notable but non-statistically significant difference (P = 0.07) for those who received intubation in theatre (RR 0.41, 95% CI 0.32 to 0.54, I^2^ = 19%) compared to those who received it outside of theatre (RR 0.68, 95% CI 0.42 to 1.09, I^2^ = 39%). Similarly, there was a non-statistically significant (P = 0.07) reduction in risk for the use of all techniques combined for individuals who are obese (RR 0.25, 95% CI 0.13 to 0.46; I^2^ = 0%) compared to individuals who were non-obese (RR 0.47, 95% CI 0.35 to 0.62). When comparing an expert and non-expert there was notable variation and non-significant difference for individuals who were non-experts (RR 0.62, 95% CI 0.32 to 1.18; I^2^ = 60%). However, there was a statistically significant reduction for those who were deemed to be expert incubators (RR 0.41, 95% CI 0.33 to 0.50; I2 = 0%). Although the difference between the two groups were non-statistically significant (P = 0.24).

### Commentary

This review was assessed using the AMSTAR 2 critical appraisal tool for systematic reviews (Shea et al 2017), and 13 out of the 16 criteria was deemed to be satisfactory (seen in Table 1). The criteria assessed as being non-satisfactory were regarding justification for study design selection, screening and data extraction. Where the study design inclusion criteria were not justified the inclusion of RCTs only (parallel, crossover and cluster) is logical based upon the objectives of the review and the large number of RCTs in this area. Regarding the lack of clarity in screening and single reviewer data extraction, it is possible that errors may have occurred within these processes. However, it can be concluded that the systematic review offers a thorough synthesis of the relevant studies concerning the research question.

When interpreting these findings in the context of pre-hospital care, it is important to note that most studies were undertaken within elective surgery and only six studies were undertaken in the pre-hospital setting. Additionally, three of the pre-hospital studies were not included in the meta-analysis as they were deemed to be outliers for the outcome of failed intubation. As there were notable high levels of failed intubation compared to the other included studies. Furthermore, there was some evidence to suggest that the environment was an important moderating factor with a nonsignificant reduction in risk being observed when comparing failed intubation in theatre compared to outside the theatre. Although there was some evidence to suggest that when performed on individuals with difficult airway features there may be an increase benefit compared to individuals with no difficulties. Therefore, when considering these findings, the certainty within the estimates provided in this review may be lower than what’s presented due to the reduced applicability of the population within the included studies to practice (Gowens et al 2011). This could be argued as being so great as to the extent where the evidence may be possibly downgraded once or twice.

However, there was “moderate certainty evidence” that all three techniques help to clinically and statistically reduce the risk of both failed intubation and Cormack-Lehane views Grade 3 or 4 being obtained. There was also “low certainty evidence” that all three techniques slightly help to increase the risk of successful first pass attempt. Suggesting that regardless of the type of videolaryngoscope used, there is an improvement in these outcomes. For the outcome of hypoxia only channelled video laryngoscopy and Macintosh-style video laryngoscopy demonstrated a statistical and clinically significant reduction based upon “moderate certainty evidence”. Furthermore, within this review only hyperangulated video laryngoscopy demonstrated a clinical and statistically significant reduction in oesophageal intubation. This may be because the hyperangulated video laryngoscopy realises the full potential of integrated camera technology by employing exaggerated curves within design to ‘look around’ the airway to optimise views of the laryngeal structures. There appears to be increasing recognition of the role of video laryngoscope within airway management practices. As indicative examples, the Difficult Airway Society state that video laryngoscopy should be immediately available wherever intubation is done and that anaesthetists should be trained in the technique (Frerk et al 2015). Furthermore, the National Institute of Health and Care Excellence (NICE) recognised the increasing role of video laryngoscopy in there 2018 publication on the topic (National Institute for Health and Care Excellence 2018). Despite this translation into pre-hospital specific guidelines does not yet appear universal although there is a trending body of evidence towards their use (Pourmand et al, 2022).

Despite the observation above and the review findings presented here, there remains a need for the assessment and empirical evaluation of videolaryngoscope for endotracheal intubation in adults in the pre-hospital setting. Such assessments should ideally be undertaken as RCTs. These RCTs may want to take a mixed method approach to explore if the high rate of failed intubation is occurring within this clinical setting and why is this the case. Where applicable relevant moderating factors should be reported and analysed such as difficult airway features and experience of the clinician. If this multi-active arm comparative approach is deemed suitable within the pre-hospital field, then using the classification of the devices within this review may help to ensure inclusion and applicability for evidence synthesis in the future. As this is a continuing developing area, this Cochrane review should be considered for development into a live network meta-analysis allowing direct and indirect live comparisons to be undertaken.

#### CPD Reflective Questions

- What is the main methodological weakness of the systematic review and how may this affect the findings from this review?
- How does limited number of studies which were included from the review being undertaken prehospital care effect the confidence in the certainty estimates presented?
- What are the clinical benefits of increasing first pass success in endotracheal intubation?
- Does the finding of a reduce risk of hypoxia during endotrachael intubation have direct relevance to pre-hospital, paramedic practice of the technique?

## Figures and Tables

**Table 1:** Critical appraisal of the review by Hansel et al (2022)

AMSTAR 2 items	Responses
1. Did the research questions and inclusion criteria for the review include the components of PICO?	Yes − The methods section of the systematic review outlined the PICO elements.
2. Did the report of the review contain an explicit statement that the review methods were established prior to the conduct of the review and did the report justify any significant deviations from the protocol?	Yes − As this was an update of a previous review a pre-existing systematic review method was already established. Two post-hoc analyses were undertaken for both sensitivity and subgroups analysis. But this was justified to statistically.
3. Did the review authors explain their selection of the study designs for inclusion in the review?	No − No justification for study design was given.
4. Did the review authors use a comprehensive literature search strategy?	Yes - A multi-database search was undertaken with relevant terms.
5. Did the review authors perform the study selection in duplicate?	No −. Where it is stated that two reviewers carried out the study selection process it is not indicated specific that this was carried out independently
6. Did the review authors perform data extraction in duplicate?	No - A verification process was carried out, but no agreement scores were given. After this verification process data extraction was undertaken by a single reviewer.
7. Did the review authors provide a list of excluded studies and justify the exclusions?	Yes - All excluded studies at full paper screening were justified.
8. Did the review authors describe the included studies in adequate details?	Yes − All main Pico variables were described.
9. Did the review authors use a satisfactory technique for assessing the risk of bias in the individual studies that were included in the review?	Yes − They use the Cochrane ROB one tool. But they did not carry this out at an outcome level.
10. Did the review authors report on the sources of funding for the studies included in the review?	Yes − All included studies funding sources were reviewed.
11. If meta-analysis was performed did the review authors use appropriate methods for statistical combination of results?	Yes - A random effects meta-analysis was used. We are clear justification of why this approach was appropriate based upon the intervention.
12. If meta-analysis was performed did the review authors assess the potential impact of RoB in individual studies on the results of the meta-analysis or other evidence synthesis?	Yes - But this was only carried out for the primary outcome of failed intubation.
13. Did the review authors account for RoB in individual studies when interpreting/discussing the results of the review?	Yes − The risk of bias was considered when establishing the certainty in the estimates given within the grade table.
14. Did the review authors provide a satisfactory explanation for and discussion of, any heterogeneity observed in the results of the review?	Yes −Some of the heterogeneity was explored for the combining of all techniques. However further exploration of the heterogeneity in the primary comparisons were using Meta-Regression was warranted.
15. If they performed quantitative synthesis did the review authors carry out an adequate investigation of publication bias (small study bias) and discuss its likely impact on the results of the review?	Yes − Both visual inspection of funnel plot and additional analyses were undertaken.
16. Did the review authors report any potential sources of conflict of interest, including any funding they received for conducting the review?	Yes - The authors declared no competing interests

## Key Points

- The Cochrane review by Hansel et al (2022) has limited direct applicability to pre-hospital care due to the limited number of studies which took place within this field.
- There is moderate certainty evidence that video laryngoscopy helps to clinically and statistically reduce the risk of failed intubation and Cormack-Lehane grade 3 or 4 occurring compared to direct laryngoscopy for adults undergoing tracheal intubation.
- There is moderate certainty evidence that channelled video laryngoscopy and Macintosh-style video laryngoscopy reduces the risk of hypoxia compared to direct laryngoscopy for adults undergoing tracheal intubation in anaesthetic settings.
- There is low certainty evidence that video laryngoscopy helps to slightly increase the risk of successful first attempt.
